# UNDERSTANDING “COMMUNITIES” IN COMMUNITY-BASED SENIOR HOUSING MODELS: A SCOPING REVIEW

**DOI:** 10.1093/geroni/igad104.3678

**Published:** 2023-12-21

**Authors:** Sojung Park, Jihye Baek, Byeongju Ryu, Ahra Ko, Jeungkun Kim, Takashi Amano, Hyeji Kim

**Affiliations:** Washington University in Saint Louis, St. Louis, Missouri, United States; Washington University in St. Louis, St. Louis, Missouri, United States; Boston College, Boston, Massachusetts, United States; Yonsei University, Seoul, Republic of Korea; Kangnam University, Yongin, Geyonggi, Republic of Korea; Rutgers University, Newark, New Jersey, United States; Good Neighbors Mirae Foundation, Seoul, Republic of Korea

## Abstract

Various community-based senior housing models have been developed to enhance social engagement and communal living among older adults, alongside their pursuit of independence and later life well-being. However, a comprehensive understanding of the efficacy of these housing models in cultivating “communities” which are integral to sustaining social ties in later life, remains limited. This scoping review investigated the conceptualization, establishment, and management of communities within diverse housing models, with an emphasis on community-related constructs. This scoping review examined empirical, peer-reviewed studies sourced from PsycINFO, CINAHL, MEDLINE, Scopus, and PubMed, resulting in the inclusion of twenty-one articles. Through thematic analysis, three predominant themes emerged: 1) residents’ well-being and lived experiences within the community (n=13), 2) engagement in communal activities and social programs (n=4), and 3) the spatial design and physical aspects of the community (n=4). We found that the way communities are defined and conceptualized differed across housing models. Also, residents’ individual characteristics (e.g., gender, age, income, health status) influenced their community lives and experiences. Community experiences were often conceptualized as a “sense of community” and “place attachment.” The majority of research focused on the experiences of residents’ communal living and their well-being. Still, some studies focused on how social and physical environments supported and facilitated community lives. This study underscores the need for further research into the dynamics of communities within diverse senior housing facilities. Also, more studies should be conducted on how those communities are formed and maintained to support older adults’ successful social lives in the community.

